# Exploring the Therapeutical Potential of *Asparagopsis armata* Biomass: A Novel Approach for Acne Vulgaris Treatment

**DOI:** 10.3390/md22110489

**Published:** 2024-10-30

**Authors:** Adriana P. Januário, Carina Félix, Rafael Félix, Katie Shiels, Patrick Murray, Patrícia Valentão, Marco F. L. Lemos

**Affiliations:** 1 MARE—Marine and Environmental Sciences Centre, ARNET—Aquatic Research Network Associated Laboratory, ESTM, Instituto Politécnico de Leiria, 2520-641 Peniche, Portugal; carina.r.felix@ipleiria.pt (C.F.); rafaelfariafelix95@gmail.pt (R.F.); 2 LAVQ—Associated Laboratory for Green Chemistry, REQUIMTE—Network of Chemistry and Technology, Faculdade de Farmácia, Universidade do Porto, 4050-313 Porto, Portugal; valentao@ff.up.pt; 3 LIFE—Health and Wellbeing Biosciences Research Institute, Shannon Applied Biotechnology Centre, Technological University of the Shannon, Moylish Park, V94 E8YF Limerick, Ireland; katie.shiels@tus.ie (K.S.); patrick.murray@tus.ie (P.M.)

**Keywords:** *Asparagopsis armata*, acne vulgaris, bioactive compounds, fatty acids, hydroethanolic extraction, Rhodophyta, seaweed extracts, skincare

## Abstract

Acne vulgaris, a high-prevalence skin condition afflicting people, persists as a significant challenge in the absence of effective treatments and emerging antibiotic resistance. To address this pressing concern, exploration of innovative approaches is of the utmost importance. *Asparagopsis armata*, an invasive red seaweed renowned for its diverse array of bioactive compounds, emerges as a promising candidate. This study seeks to elucidate the potential utility of *A. armata* biomass in the treatment of acne vulgaris. Crude extracts were obtained through solid–liquid extraction, and fractions were obtained using liquid–liquid extraction. The analyzed bioactivities included antioxidant, antimicrobial, and anti-inflammatory. Also, chemical characterization was performed to identify free fatty acids and compounds through LC-MS and elements. The present findings unveil compelling attributes, including anti-*Cutibacterium acnes* activity, cytotoxic and non-cytotoxic effects, antioxidant properties, and its ability to reduce nitric oxide production with consequent anti-inflammatory potential. Additionally, chemical characterization provides insights into its mineral elements, free fatty acids, and diverse compounds. The observed antimicrobial efficacy may be linked to halogenated compounds and fatty acids. Cytoprotection appears to be associated with the presence of glycerolipids and glycosylated metabolites. Furthermore, its antioxidant activity, coupled with anti-inflammatory properties, can be attributed to phenolic compounds, such as flavonoids. This study underscores the potential of *A. armata* as a natural ingredient in skincare formulations, offering an important contribution to the ongoing battle against acne vulgaris.

## 1. Introduction

Acne poses a wide array of psychological challenges for individuals, leading to reduced self-confidence, feelings of depression, heightened anxiety, and difficulties in navigating interpersonal relationships and professional environments [1]. Acne vulgaris (AV) ranks as the eighth most prevalent skin condition worldwide, with an estimated prevalence of 9.38% across all age demographics. This disease affects roughly 85% of adolescents and continues into adulthood for approximately 50% of individuals [2]. Acne vulgaris is characterized by five interrelated pathophysiological factors, including hormonal dysregulation, increased sebum production, epidermal keratinocytes differentiation, *Cutibacterium acnes* hyperproliferation and/or dysbiosis, and inflammatory response (reviewed in [3]). Dermatologists recommend various treatment options for AV, including antibiotics, such as clindamycin and erythromycin, benzoyl peroxide, and retinoids [4]. However, these treatments are not without challenges: antibiotic resistance has become a concern, limiting their effectiveness and use [5]; benzoyl peroxide has been associated with allergic contact dermatitis and facial edema [6]; and retinoids can cause excessive dryness of the skin [7]. While treatments for AV can sometimes be effective, the chronic nature of the condition often results in recurrences [8]. Hence, it is imperative to uncover novel therapeutic agents capable of effectively addressing the disease, achieving successful treatment outcomes, maintaining acne remission, minimizing the risk of relapse, and doing so without inducing significant side effects.

Macroalgae, as sessile marine organisms, inhabit an ever-changing environment, where they must cope with fluctuations in both biotic and abiotic factors, including light, nutrients, salinity, temperature, and predation [9]. To thrive and survive in such conditions, seaweeds have developed the ability to synthesize specific compounds that enable them to adapt and respond to these variations [10]. Rhodophyta are known producers of anti-inflammatory metabolites, such as mycosporine-like amino acids (MAAs) [11], phycocyanin, allophycocyanin, carotenoids [12], and porphyrins [13]. Also, antimicrobial substances like terpenes, phenolic compounds, sterols, polysaccharides, and fatty acids [14] are synthesized by those macroalgae. The red seaweed *Asparagopsis armata* Harvey 1855, native to the Western Australian coast [15], is now widely distributed in the Portuguese coast [16]. Ecotoxic metabolites produced by the seaweed offer an ecological advantage by disrupting the growth of competing algae, leading to the formation of exclusive seaweed-dominated areas, diminishing the overall diversity of species in the habitat [17,18]. Despite its negative ecological impact, *A. armata* has been commercially farmed in Ireland since the 1990s to extract bioactive molecules that are applied in some cosmetics [19] (e.g., Algaran™ Organic Day Face Cream with hydrating properties), or hand-picked at invaded habitats, such as Azores, for the same purposes (e.g., BodyOcean^®^, a regenerator facial cream).

Counting on the successful incorporation of these extracts into skincare formulations and leveraging their diverse compounds and bioactivities capable of addressing various stages of acne vulgaris, this study endeavors to evaluate the anti-acne potential of an industry-compatible hydroethanolic crude extract of *A. armata*, along with its liquid–liquid partitions, obtained through common solvents and an industry-friendly extraction process.

## 2. Results

### 2.1. Extraction Yield

The hydroethanolic solid–liquid crude extraction was conducted thrice using the same dried biomass of *A. armata*, resulting in three extracts with approximately identical yields (HE R1, HE R2, HE R3; 14.03% ± 0.93). Liquid–liquid sequential extraction involved combining equal amounts of the three crude extracts, followed by a second extraction, which resulted in lower yields when organic solvents were used: 2.97% for n-hexane (HEH), and 4.09% for ethyl acetate (HEEA), with a higher yield of 92.94% for the aqueous remnant (HEW) (Table 1).

### 2.2. Antioxidant Activity

**Figure 1:**
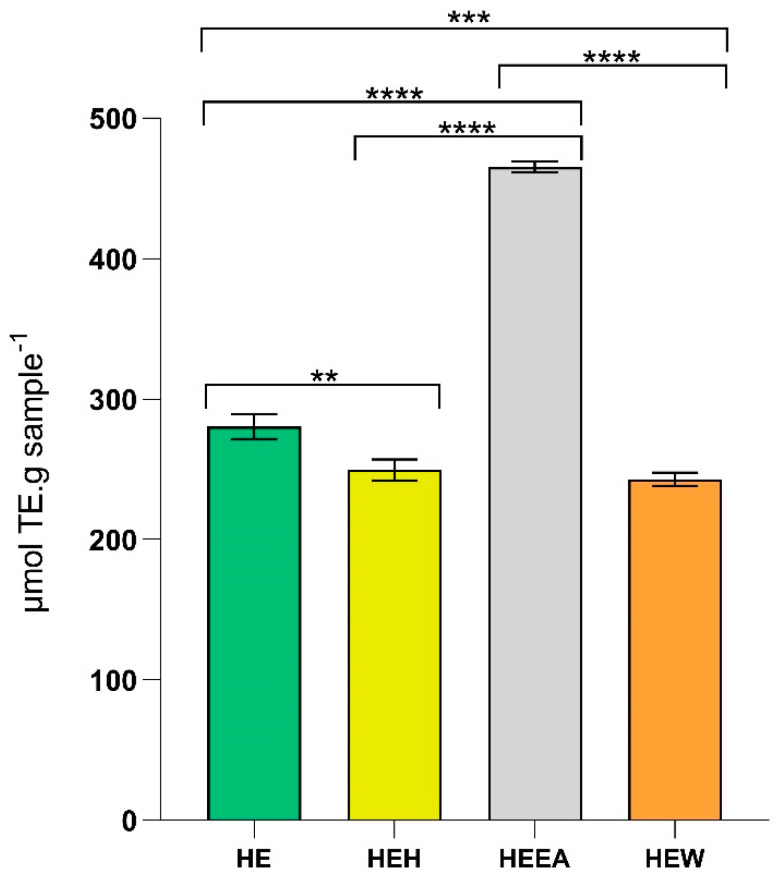
Antioxidant capacity revealed by the ORAC assay expressed as µmol of Trolox equivalents (TE) per gram of *Asparagopsis armata* extract (HE—hydroethanolic solid–liquid crude extraction) and fractions (HEH—liquid–liquid sequential extraction from HE using n-hexane; HEEA—liquid–liquid sequential extraction from HE using ethyl acetate; HEW—aqueous remnant of the liquid–liquid sequential extraction from HE). One-way ANOVA was performed, followed by Tukey’s multiple comparisons test, to assess the significant differences in the antioxidant activity obtained between treatments. ** *p* < 0.01, *** *p* < 0.001, **** *p* < 0.0001. The reported values represent the mean ± SD from three independent experiments.

The oxygen radical absorbance capacity (ORAC) assay was used to evaluate the antioxidant activity of the *A. armata* extracts and fractions. The HEEA fraction demonstrated the highest ORAC activity at 1 mg·mL^−1^ (465.18 µmol TE·g sample^−1^; *p* < 0.0001), while the HEW fraction showed the lowest activity at the same concentration (242.84 µmol TE·g sample^−1^; *p* < 0.0001 compared with HEEA fraction, *p* < 0.001 compared with HE, and no differences compared with HEH). The HEH fraction also exhibited lower activity (249.47 µmol TE·g sample^−1^; *p* < 0.0001 compared with HEEA fraction, *p* < 0.01 compared with HE, and no differences compared with HEW) (Figure 1).

### 2.3. Antimicrobial Susceptibility Testing (AST)

**Figure 2:**
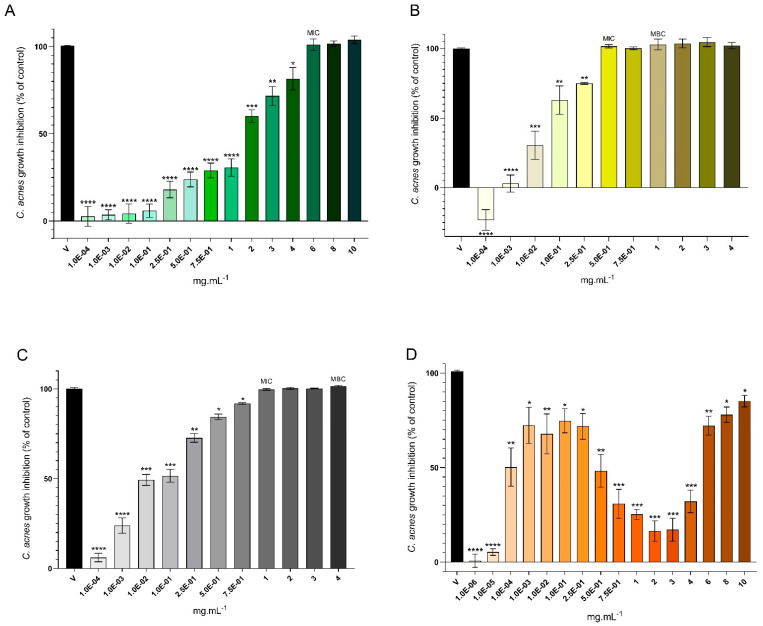
Antimicrobial activity against *Cutibacterium acnes* of *Asparagopsis armata* extract: (**A**)—HE (hydroethanolic solid–liquid crude extraction) and its fractions; (**B**)—HEH (liquid–liquid sequential extraction from HE using n-hexane); (**C**)—HEEA (liquid–liquid sequential extraction from HE using ethyl acetate); (**D**)—HEW (aqueous remnant of the liquid–liquid sequential extraction from HE), at the following concentrations: HE—1.0 × 10^−4^, 1.0 × 10^−3^, 1.0 × 10^−2^, 1.0 × 10^−1^, 2.5 × 10^−1^, 5.0 × 10^−1^, 7.5 × 10^−1^, 1, 2, 3, 4, 6, 8, and 10 mg·mL^−1^; HEH and HEEA—1.0 × 10^−4^, 1.0 × 10^−3^, 1.0 × 10^−2^, 1.0 × 10^−1^, 2.5 × 10^−1^, 5.0 × 10^−1^, 7.5 × 10^−1^, 1, 2, 3, and 4 mg·mL^−1^; and HEW—1.0 × 10^−6^, 1.0 × 10^−5^, 1.0 × 10^−4^, 1.0 × 10^−3^, 1.0 × 10^−2^, 1.0 × 10^−1^, 2.5 × 10^−1^, 5.0 × 10^−1^, 7.5 × 10^−1^, 1, 2, 3, 4, 6, 8, and 10 mg·mL^−1^. The minimum inhibitory concentration (MIC) and minimum bactericidal concentration (MBC) were also evaluated and are discriminated against the respective concentrations. One-way ANOVA was performed, followed by Dunnett’s multiple comparisons test, to assess the significant differences in *C. acnes* growth inhibition in the presence of the extract/fractions compared to the inhibition control (V—Vancomycin at 4 μg·mL^−1^). * *p* < 0.05, ** *p* < 0.01, *** *p* < 0.001, **** *p* < 0.0001. The reported values represent the mean ± SD from six independent experiments.

The antimicrobial activity of *A. armata* extracts and fractions was tested against the bacterium that is mainly involved in acne vulgaris disease: *Cutibacterium acnes* (DSM-1897) (Figure 2). All concentrations of the crude extract led to growth inhibition, achieving MIC (Minimum Inhibitory Concentration) at 6 mg·mL^−1^, while no MBC (Minimum Bactericidal Concentration) was found within the tested concentrations (HE; Figure 2A). Compared with the inhibition control (vancomycin), 6, 8, and 10 mg·mL^−1^ showed no significant differences (Figure 2A). Organic fractions showed the most potent antimicrobial activity (Figure 2B,C) compared to the growth inhibition effect of HE and HEW (Figure 2A,D). The n-hexane fraction had a MIC at 5.0 × 10^−1^ mg·mL^−1^ and MBC at 1 mg·mL^−1^, wherein this fraction displayed the strongest *C. acnes* growth inhibition (Figure 2B), followed by HEEA (Figure 2C), presenting MIC at 1 mg·mL^−1^ and MBC at 4 mg·mL^−1^. The MIC and MBC values were not found for HEW (Figure 2D), but at 1.0 × 10^−4^ mg·mL^−1^, *C. acnes* growth inhibition was reported as 50.19% (Figure 2D).

### 2.4. Cytotoxicity and Anti-Inflammatory Activity

#### 2.4.1. Cytotoxicity Evaluation

**Figure 3:**
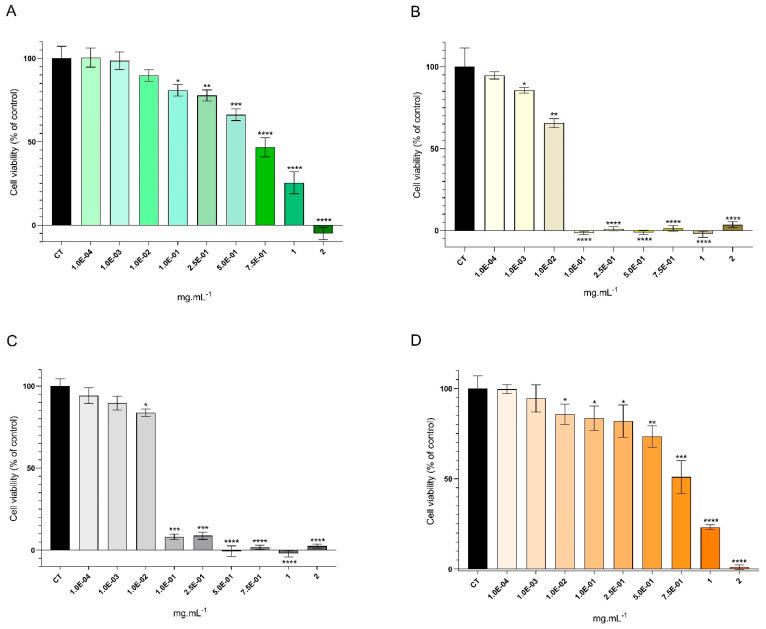
Viability of the HaCaT cell line exposed to *Asparagopsis armata* extract: (**A**)—HE (hydroethanolic solid–liquid crude extraction) and its fractions; (**B**)—HEH (liquid–liquid sequential extraction from HE using n-hexane); (**C**)—HEEA (liquid–liquid sequential extraction from HE using ethyl acetate); (**D**)—HEW (aqueous remnant of the liquid–liquid sequential extraction from HE), at the following concentrations: HE and HEW—1.0 × 10^−4^, 1.0 × 10^−3^, 1.0 × 10^−2^, 1.0 × 10^−1^, 2.5 × 10^−1^, 5.0 × 10^−1^, 7.5 × 10^−1^, 1, and 2 mg·mL^−1^; HEH and HEEA—1.0 × 10^−4^, 1.0 × 10^−3^, 1.0 × 10^−2^, 1.0 × 10^−1^, 2.5 × 10^−1^, 5.0 × 10^−1^, 7.5 × 10^−1^, 1, and 2 mg·mL^−1^. One-way ANOVA was performed, followed by Dunnett’s multiple comparisons test, to assess the significant differences in HaCaT cell viability in the presence of the extract/fractions compared to the growth control (CT, non-treated cells). * *p* < 0.05, ** *p* < 0.01, *** *p* < 0.001, **** *p* < 0.0001. The reported values represent the mean ± SD from at least four independent experiments.

**Figure 4:**
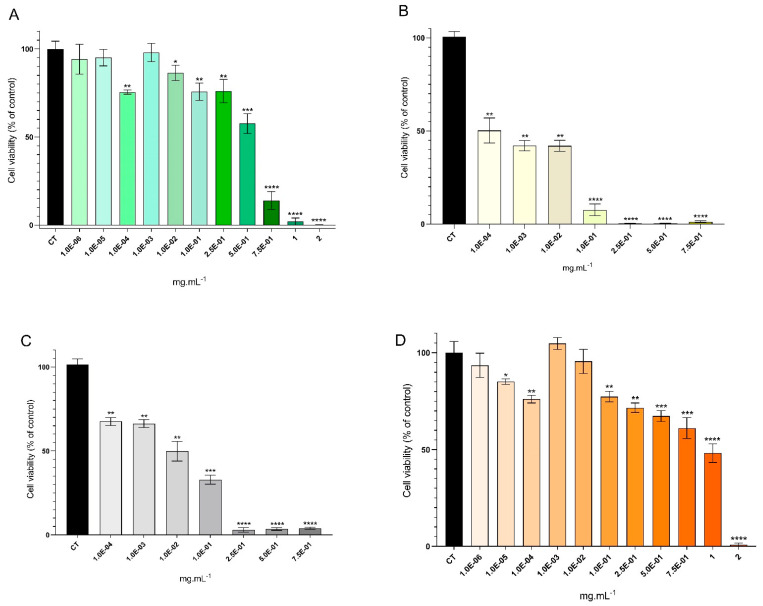
Viability of the RAW 264.7 cell line exposed to *Asparagopsis armata* extract: (**A**)—HE (hydroethanolic solid–liquid crude extraction) and its fractions; (**B**)—HEH (liquid–liquid sequential extraction from HE using n-hexane); (**C**)—HEEA (liquid–liquid sequential extraction from HE using ethyl acetate); (**D**)—HEW (aqueous remnant of the liquid–liquid sequential extraction from HE), at the following concentrations: HE and HEW—1.0 × 10^−6^, 1.0 × 10^−5^, 1.0 × 10^−4^, 1.0 × 10^−3^, 1.0 × 10^−2^, 1.0 × 10^−1^, 2.5 × 10^−1^, 5.0 × 10^−1^, 7.5 × 10^−1^, 1, and 2 mg·mL^−1^; HEH and HEEA—1.0 × 10^−4^, 1.0 × 10^−3^, 1.0 × 10^−2^, 1.0 × 10^−1^, 2.5 × 10^−1^, 5.0 × 10^−1^, and 7.5 × 10^−1^ mg·mL^−1^. One-way ANOVA was performed, followed by Dunnett’s multiple comparisons test, to assess the significant differences in RAW 264.7 cell viability in the presence of the extract/fractions compared to the growth control (CT, non-treated cells). * *p* < 0.05, ** *p* < 0.01, *** *p* < 0.001, **** *p* < 0.0001. The reported values represent the mean ± SD from at least four independent experiments.

The viability of both HaCaT and RAW 264.7 cell lines exposed to *A. armata* extracts and fractions was evaluated using the MTT assay (Figure 3 and Figure 4, respectively). Overall, diminished concentrations of the extract and fractions were associated with increased levels of cellular viability.

The exposure of HaCaT cells to the crude extract revealed no significant differences in comparison to control (non-treated cells), from a concentration of 1.0 × 10^−4^ mg·mL^−1^ to 1.0 × 10^−2^ mg·mL^−1^. Also, increased extract concentration correlated with elevated cytotoxicity, and, as the concentration increased, the statistical differences became more pronounced, starting at 1.0 × 10^−1^ mg·mL^−1^ (Figure 3A). A comparable trend was observed in cells exposed to the HEW fraction, although with a discrepancy observed at lower concentrations, wherein the viable concentrations were 1.0 × 10^−3^ mg·mL^−1^ and 1.0 × 10^−4^ mg·mL^−1^ (Figure 3D). On the other hand, the organic fraction HEH led to lower cell viability (Figure 3B), with statistically significant differences observed starting at a concentration of 1.0 × 10^−3^ mg·mL^−1^. Moreover, concentrations ranging from 1.0 × 10^−1^ mg·mL^−1^ to 2 mg·mL^−1^ were highly cytotoxic, wherein minimal or no viability was recorded. A similar pattern was noted in cells exposed to the HEEA fraction, although with a disparity where statistically significant differences began at a higher extract concentration (1.0 × 10^−2^ mg·mL^−1^). Also, heightened extract concentration was associated with increased cytotoxicity, and with an increase in concentration, the statistical differences became more pronounced. To summarize, HE extract was the treatment leading to greater cell viability: 100.4% at 1.0 × 10^−4^ mg·mL^−1^, 98.5% at 1.0 × 10^−3^ mg·mL^−1^, and 89.7% at 1.0 × 10^−2^ mg·mL^−1^ (Figure 3A). On the contrary, the treatment with the HEH fraction negatively affected cell viability, and values around 0% were found from a concentration of 1.0 × 10^−1^ mg·mL^−1^ up to 2 mg·mL^−1^ (Figure 3B).

The same patterns were observed for RAW 264.7 cell viability (Figure 4), but this cell line revealed greater sensitivity compared to HaCaT when it came to the induced toxicity of *A. armata* extract/fractions. The most viable treatment was achieved when cells were exposed to the HE extract (Figure 4A), showing no significant differences at 1.0 × 10^−4^, 1.0 × 10^−3^, and 1.0 × 10^−2^ mg·mL^−1^; the lowest cellular viability was once again attained using the HEH fraction (Figure 4B) and HEEA fraction (Figure 4C), with all concentrations tested showing differences compared to the control of non-treated cells. Likewise, elevated extract concentration was found to correlate with heightened cytotoxicity, with the emergence of more pronounced statistical differences in all cases. Cells exposed to the HEW fraction (Figure 4D) exhibited a comparable trend to the HE results (Figure 4A), although a discrepancy was noted at lower concentrations, wherein higher values of cell viability were identified at 1.0 × 10^−3^ mg·mL^−1^ and 1.0 × 10^−4^ mg·mL^−1^ (Figure 4D). Conversely, the HEH organic fraction demonstrated greater toxicity, with significant statistical differences detected at a concentration of 1.0 × 10^−3^ mg·mL^−1^ (Figure 4B). Additionally, concentrations ranging from 1.0 × 10^−1^ mg·mL^−1^ to 2 mg·mL^−1^ exhibited elevated cytotoxicity, where minimal or no viability was recorded. To summarize, HE extract was the treatment that led to more viable cells: 97.9% at 1.0 × 10^−3^ mg·mL^−1^, 95.1% at 1.0 × 10^−5^ mg·mL^−1^, and 94.2% at 1.0 × 10^−6^ mg·mL^−1^ (Figure 4A). Also, HEH fraction was the treatment leading to lower values of cell viability, where the maximum recorded was 50.2% at 1.0 × 10^−4^ mg·mL^−1^ (Figure 4B).

#### 2.4.2. Nitric Oxide (NO) Production

**Figure 5:**
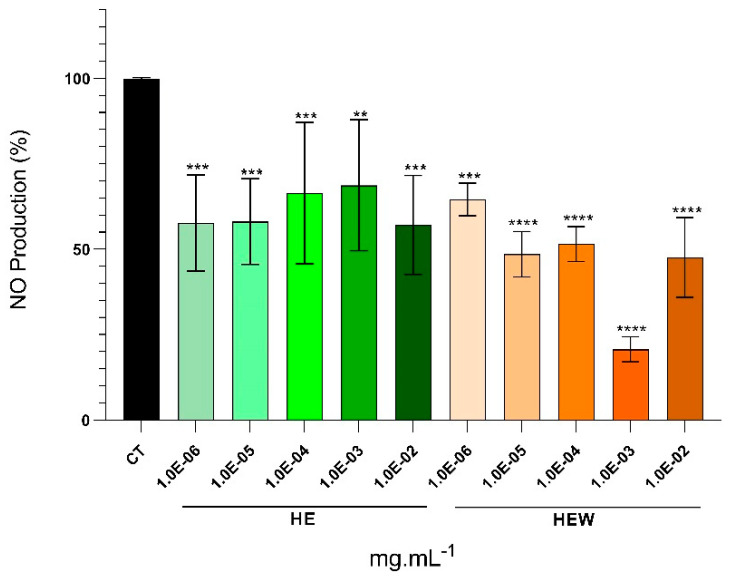
Nitric oxide assay performed on RAW 264.7 cell line using concentrations of 1.0 × 10^−6^, 1.0 × 10^−5^, 1.0 × 10^−4^, 1.0 × 10^−3^, and 1.0 × 10^−2^ mg·mL^−1^ of the *Asparagopsis armata* crude extract (HE—hydroethanolic solid–liquid crude extraction) and aqueous fraction (HEW—aqueous remnant of the liquid–liquid sequential extraction from HE) to assess the anti-inflammatory potential. Control (CT) represents LPS-stimulated cells (1.5 µg·mL^−1^). One-way ANOVA was performed, followed by Dunnett’s multiple comparisons test, to assess the significant differences in NO production with LPS-stimulated cells followed by treatment with *A. armata* extract and fraction compared to the control (LPS-stimulated cells). ** *p* < 0.01, *** *p* < 0.001, **** *p* < 0.0001. The reported values represent the mean ± SD from five independent experiments, and the error bars represent the standard deviation.

Concentrations with over 75% macrophage cell viability were employed to assess nitric oxide production and, consequently, the anti-inflammatory potential of *A. armata* biomass (Figure 5). This decision led to the exclusion of HEH and HEEA fractions, and the assay only included treatments with HE and HEW (Figure 4). The NO production of RAW 264.7 cells challenged with LPS and subjected to HE and HEW was reduced for all tested concentrations compared to the control (LPS-stimulated cells with no treatment) (Figure 5). Moreover, the highest reduction in NO production occurred at 1.0 × 10^−3^ mg·mL^−1^ of the HEW fraction (79.32%, *p* < 0.0001) and at 1.0 × 10^−2^ mg·mL^−1^ of the crude extract (42.89%, *p* < 0.0001) (Figure 5).

### 2.5. Chemical Characterization

#### 2.5.1. Free Fatty Acids

**Figure 6:**
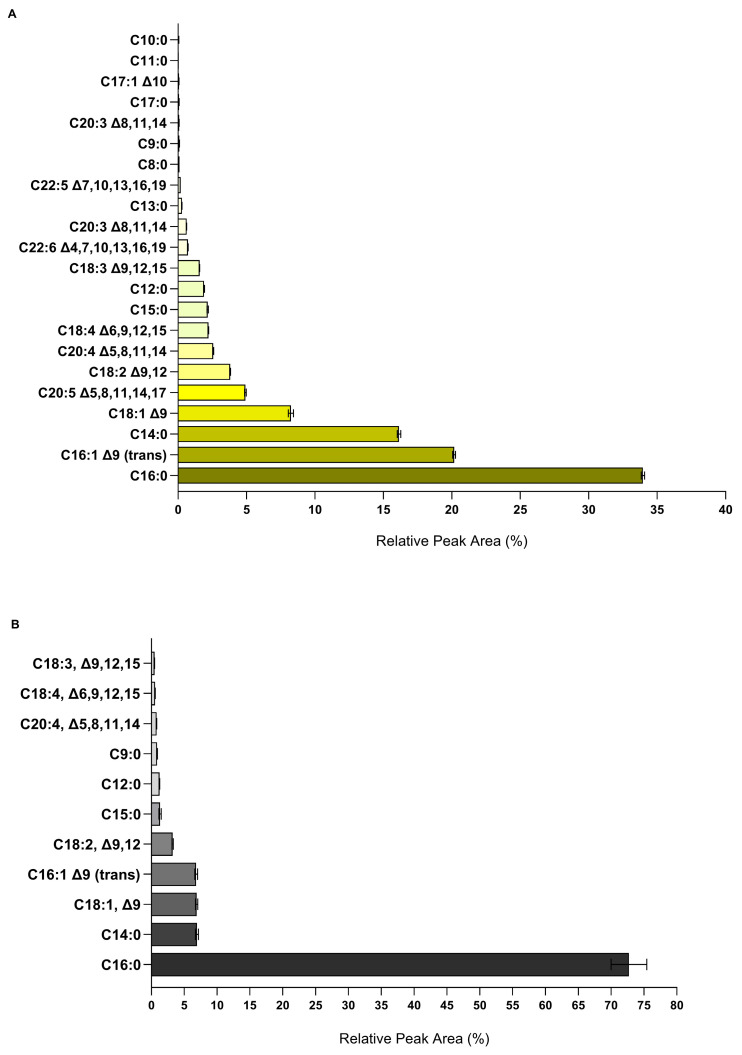
Relative peak area (%) of individual free fatty acids relative to the total free fatty acids content. Analysis via LC-MS was performed at 1 mg·mL^−1^ (**A**) HEH and (**B**) HEEA fractions of *Asparagopsis armata*. The error bars represent the standard deviation.

Free fatty acid analysis was conducted with the two organic fractions of *A. armata* extract due to the nature of the extraction solvent, wherein this type of biomolecule was expected, and not with the aqueous fraction, and it is presented in Figure 6. Concerning the free fatty acid profile of the HEH fraction (Figure 6A), palmitic acid (C16:0) dominates (33.96%), followed by palmitelaidic acid (16:1, Δ9 (trans); 20.17%), myristic acid (C14:0; 16.14%), oleic acid (18:1, Δ9; 8.24%), eicosapentaenoic acid (20:5, Δ5,8,11,14,17; 4.92%), linoleic acid (18:2, Δ9,12; 3.81%), arachidonic acid (20:4, Δ5,8,11,14; 2.57%), stearidonic acid (18:4, Δ6,9,12,15; 2.23%), pentadecylic acid (C15:0; 2.17%), lauric acid (C12:0; 1.90%), linolenic acid (18:3, Δ9,12,15; 1.57%), and 11 others at lower proportions, contributing to a total of 22 fatty acids. The analysis of the HEEA fraction (Figure 6B) allowed for the identification of 11 free fatty acids, with palmitic acid being the most prominent, accounting for 72.71% of the total free fatty acids in this fraction, followed by myristic acid (C14:0; 6.94%), oleic acid (18:1, Δ9; 6.88%), palmitelaidic acid (16:1, Δ9 (trans); 6.82%), and linoleic acid (18:2, Δ9,12; 3.23%). Overall, the HEH fraction exhibits greater diversity in free fatty acids, approximately twice that of the HEEA fraction. Both fractions highlight the prevalence of palmitic acid, as well as considerable amounts of myristic, palmitelaidic, oleic, and linoleic acids (Figure 6A,B).

#### 2.5.2. Compound Screening 

The compounds tentatively identified via LC-MS in the different fractions of *A. armata* are reported in Table 2 (HEH), Table 3 (HEEA), and Table 4 (HEWm). Among the fractions, HEEA exhibited a higher number of identified compounds (12), followed by HEH (8), while HEWm demonstrated a comparatively lower count (3). Phospholipids were the molecules that were most frequently found across all the *A. armata* fractions, specifically phosphatidic acids (PAs), phosphatidylinositols (PIs), and phosphatidylglycerols (PGs) (Table 2, Table 3 and Table 4). The HEH fraction showed to possess the halogenated compound Malyngamide L (Table 2), and both HEEA and HEWm fractions (Table 3 and Table 4) contained glycerolipids.

#### 2.5.3. Element Profile

The extract and fractions’ element fingerprinting was conducted to determine the element composition. *A. armata* extracts and fractions primarily contained high amounts of bromine (Br), followed by chlorine (Cl) and sodium (Na) (Table 5). Most of the quantified elements were found in the crude extract (HE) and its aqueous fraction (HEW) (Table 5).

## 3. Discussion

In recent years, the demand for eco-friendly cosmetics has grown significantly, driven by a global push toward sustainability [20]. The natural skincare market is expected to double from USD 9.9 billion in 2021, to USD 20.4 billion by 2030, highlighting its crucial role in the cosmetic industry. Exploring invasive species like *A. armata* for cosmetic applications offers a cost-effective and eco-friendly sourcing opportunity [21]. *A. armata* is rich in bioactive compounds with various benefits, making it a promising option for sustainable acne vulgaris treatment through functional cosmetics or dermocosmetics [22]. This marine resource holds potential for developing sustainable options for AV treatment, including creating functional cosmetics, cosmeceuticals, or dermocosmetics for daily skincare, since acne vulgaris is the eighth most prevalent dermatological issue globally [23].

Over the past few years, a few cosmetic products targeting acne have begun incorporating extracts from *A. armata* into their ingredients. However, the publicly available information on the products does not specify the type of extract(s) used or the specific function it confers to the product and, consequently, to the skin and to the management of a given disease. It is worth noting that these products often include additional antimicrobial and/or anti-sebum ingredients alongside the *A. armata* extract(s). Nevertheless, considering the beneficial bioactivities demonstrated by extracts of *A. armata* against certain factors related to acne vulgaris (such as the inhibition of *C. acnes* by hydrothermal and supercritical extracts [24] and the anti-inflammatory activity of a derived polysaccharide [25]), the present study focused on understanding how simple and cost-effective extracts could be of use to fight the disease and depicting their modes of action.

Biomass extraction parameters were set to achieve high yields, cost effectiveness, and maximum compound recovery, aligning with industry standards. This can be achieved using ambient temperature to conserve energy and prevent compound degradation, with water and/or widely approved organic solvents used as extraction media [26,27]. A hydroethanolic solid–liquid crude extraction was performed, yielding 14.03% (±0.93)—a higher yield compared to a previous study that used similar extraction methodologies (reported yields between 1.3% and 1.6%) [28]. Secondary liquid–liquid extractions resulted in lower yields with organic solvents but significantly higher ones with aqueous extraction. Solvent selection aimed to extract low-molecular-weight carbohydrates and peptides, soluble in water [29], while ethanol facilitated the extraction of compounds with a wide range of polarities, including diverse glycosides, phenolic compounds, and lipids (including fatty acids, glycerolipids, phospholipids, glycolipids, and steroids) [30,31], as confirmed by LC-MS analysis.

The HEEA fraction showed the strongest antioxidant activity in the ORAC assay (465.2 ± 3.2 µmol TE·g sample^−1^), followed by the crude extract (HE), the HEH fraction, and HEW, the latter fraction exhibiting the lowest activity (242.8 ± 3.8 µmol TE·g sample^−1^). These findings are supported by research on the antioxidant activity of red seaweeds [32], in which the ethyl acetate fraction displayed the highest levels of total phenolic content and other antioxidant assays compared to the n-hexane and dichloromethane fractions and the crude methanol extract. The authors suggest that phenolic compounds are responsible for the antioxidant activity. Indeed, the HEEA fraction was found to contain galactosylglycerol and monogalactosyldiacylglycerol, which are known to possess antioxidant activity (Table 3) [33,34]. It is important to address the fact that phenolics are not the only antioxidants in this fraction. Medium-polarity compounds, such as phospholipids, containing PUFAs are well known antioxidants [35,36], making an important contribution to the overall antioxidant capacity of this fraction. In addition to HEEA, the crude extract (HE) also showed significant antioxidant activity (280.3 ± 7.3 µmol TE·g sample^−1^; Figure 1). This activity can be attributed to the combined presence of all compounds identified in the three subsequent fractions (HEH, HEEA, and HEW; Table 2, Table 3 and Table 4, respectively). Additionally, previous studies indicate that methanolic and aqueous extracts of red algae exhibit considerable antioxidant activity, suggesting the presence of unidentified phenolic compounds that contribute to this antioxidant capacity [28,37,38]. The literature also suggests that low-molecular-weight fragments of sulphated polysaccharides may play a significant role in the antioxidant activity of these extracts [39]. In terms of activity order, the HEH fraction showed intermediate antioxidant activity (249.5 ± 6.2 µmol TE·g sample^−1^; Figure 1). This result is consistent with the hypothesis that less polar compounds, extracted with n-hexane, have a lower antioxidant contribution compared to the more polar compounds found in the HEEA fraction [40]. However, the presence of certain phospholipids, and halogenated compounds, with documented antioxidant activity may explain the observed activity for the HEH fraction [41,42]. Lastly, the HEW fraction, although exhibiting the lowest antioxidant activity (242.8 ± 3.8 µmol TE·g sample^−1^; Figure 1), still maintained a significant capacity, corresponding to approximately half of the activity of HEEA. This residual activity can be attributed to the antioxidant compounds present in the other fractions that, although in smaller quantities, remain in the aqueous fraction. The antioxidant capability of *A. armata* methanolic and aqueous extracts has already been reported [37,38]. Moreover, a UHPLC-MS qualitative analysis of another study revealed that the water extract of *A. armata* possesses phenolic compounds [28], which are expected to have been extracted by our hydroethanolic solvent even further (given their high affinity toward polar organic solvents, such as ethanol [31]). That being the case, although the LC-MS of HEWm failed to identify such variety of phenolic compounds, it is possible that they are present and responsible for part of the antioxidant activity of this fraction. Thus, the diversity of the compounds that are present in the different fractions of *A. armata*, from phenolics to phospholipids, significantly contributes to the observed antioxidant activity. The complexity of the antioxidant compound matrix highlights the importance of considering both the identified compounds and those that may be present in smaller quantities or have not yet been characterized, but which together provide strong antioxidant capacity to the different fractions and the crude extract. Oxidative processes play a crucial role in the development of AV. For instance, the stimulatory effect of keratinocytes exerted by *C. acnes* induces the production of ROS, triggering NO production and exacerbating the inflammatory response (reviewed in [3]), and therefore, there is a fundamental importance in having antioxidant compounds with the capacity to potentially control AV.

The relationship between effect and concentration does not always follow the commonly expected classic sigmoidal pattern, as shown in the antimicrobial activity of HE and HEEA curves (Figure 2A,C). Unlike those, concentration–response curves exhibiting a bell- or “U”-shaped profile (e.g., HEW; Figure 2D) and hermetic dose–response (e.g., HEH; Figure 2B) indicate more intricate biological effects, potentially involving events like multiple-binding sites, multiple mechanisms of action and targets [43], making its comprehensive analysis a challenge. An illustrative case in the literature is the concentration–response relationship for the oxacillin (OXA) product OXA-SER, which revealed a MIC value of 2.5 × 10^−4^ mg·mL^−1^ against *S. aureus*, which also displayed a distinctive pharmacodynamic pattern characterized by a U-shaped curve [44]. Some authors suggest that some drugs may display non-monotonic U-shaped curves during transition to colloidal aggregates, contrasting with monotonic sigmoidal curves in their monomeric state [43,45], and such behavior may explain the fluctuating antimicrobial activity observed in the HEW fraction (Figure 2D), since this fraction is expected to possess several oligomers. Lower concentrations may imply a monomeric state, inhibiting bacterial growth effectively, while moderate concentrations may lead to aggregate formation, reducing efficacy. At the highest concentrations (6, 8, and 10 mg·mL^−1^), the nearly 100% growth inhibition suggests a mixture of monomeric and aggregated forms, where monomers can penetrate cells via passive diffusion, while aggregates cannot. Although other authors reported non-monotonic dose–response curves in several experiments [46,47,48,49,50], this kind of behavior is yet to be completely elucidated, and more mechanistic studies are needed to address such issues. Also, synergistic effects have been proven when it comes to antimicrobial bioactive compounds [51,52], antagonistic interactions [53], and additive dose–response effects [53], which may also explain different behaviors in the antimicrobial outcomes (Figure 2).

Studies reporting the susceptibility of *C. acnes* to *A. armata* extracts are almost inexistent. In a previous work, Vega et al. [38] employed hydroethanolic (1:4, w/EtOH) and aqueous extracts of *A. armata* but found no inhibitory effect on *C. acnes* growth, which contrasts with the findings reported herein. The difference between Vega et al.’s study and ours may be due to the seasonal and geographic variability in the algae, among other factors, and this should be explored in future studies to determine the biomass factors that need to be controlled in their industrial exploitation. Nonetheless, several studies have found significant antibacterial activity in *A. armata* extracts prepared with organic solvents, including methanol-toluene [54], methanol, n-hexane, and dichloromethane [55,56], and ethanol [26]. Moreover, the predominant antibacterial properties of these algae are linked to halogenated molecules identified in a study that evaluated an apolar phase, which showed antibacterial activity [57]. Indeed, in the present study, the organic fractions contained bromine, chlorine, and iodine (Table 5), which are the indicators of larger quantities of halogenated compounds than those that were found. Nonetheless, the halogenated compound in the apolar HEH fraction (Table 2), malyngamide L, represented the most potent antibacterial fraction, with MIC at 5.0 × 10^−1^ mg·mL^−1^. Also, both fractions HEH and HEEA, had several antibacterial fatty acids (Figure 6), such as eicosapentaenoic acid (20:5 Δ5,8,11,14,17), which has been studied extensively for its strong antibacterial properties that showed significant activity against a variety of pathogens, including *Staphylococcus aureus* and *Escherichia coli*. The EPA’s antibacterial effect is attributed to its ability to disrupt bacterial cell membranes and inhibit growth [58,59]. Linoleic acid (18:2 Δ9,12) also exhibited notable antibacterial activity, and research indicates that it is effective against Gram-positive bacteria like *S. aureus*, and it has been used in formulations to enhance antimicrobial efficacy [58,59]. Myristic acid (14:0) has demonstrated antibacterial properties, particularly against Gram-positive bacteria, being effective in disrupting bacterial membranes and inhibiting microbial growth [58,59]. Oleic acid (18:1 Δ9) showed moderate antibacterial activity, with some studies indicating effectiveness against both Gram-positive and -negative bacteria, and it is often used in combination with other fatty acids to enhance antimicrobial properties [58,59]. Palmitelaidic acid (16:1 Δ9 (trans)) has been studied for its antibacterial properties, particularly against skin pathogens like *C. acnes* and *S. aureus*; however, its efficacy is lower compared to polyunsaturated fatty acids [58,59]. Nevertheless, HEW also contains other antibacterial constituents, such as glycosylated metabolites like gingerglycolipid A [60], and the glycerolipid DG (24:1(15Z)/22:6(4Z,7Z,10Z,13Z,16Z,19Z)/0:0) that contains DHA in its composition [58].

Occupying over 90% of the epidermis, keratinocytes serve both as a physical barrier and an integral component of the innate immune defense [61]. Thus, it is crucial not to negatively impact these cells when developing any type of skincare product. To our knowledge, the present study is the first to report the cytotoxicity of *A. armata* extracts in human keratinocyte cells (HaCaTs). The cytotoxicity of *A. armata* in RAW 264.7 (mouse macrophage) cells was also determined, further highlighting the sensitivity of mammalian cells to this seaweed’s components. The results indicate that lower extract/fraction concentrations lead to higher cell viability. The HaCaT cytotoxicity assay showed that both the extract and all fractions have several concentrations where cell viability is high (Figure 3), and RAW 264.7 cells showed more sensitivity, whereinboth organic fractions revealed low numbers of cell viability (Figure 4B,C). For both cell lines, HEW proved to be the fraction that showed greater cell viability across several concentrations, while HEH showed an opposite trend. Prior investigations have assessed the cytotoxicity of various Rhodophyta extracts in human keratinocytes. For instance, *Pyropia yezoensis* (Rhodophyta) methanolic extracts were tested across multiple concentrations (0.01–2 mg·mL^−1^), revealing low levels of cytotoxicity in all tested concentrations, except for cytotoxic effects at the highest concentration studied [62]. The authors suggest that carotenoids from *P. yezoensis* are cytoprotective due to their antioxidant and anti-inflammatory properties [62]. Piao et al. [63] performed ethanolic extraction using *Chondracanthus tenellus* (Rhodophyta) and found non-cytotoxic effects on HaCaT cells. Also, a hydroethanolic extract of *Kappaphycopsis cottonii* (formerly *Eucheuma cottonii*) (Rhodophyta) showed no cytotoxicity up to 200 µg·mL^−1^ in HaCaT cells [64]—a result similar to the one found in the present study. The authors indicate that the halogenated phenolic compounds usually found in red seaweeds are the main reason for cytotoxic effects [65], which may be the case regarding the high cytotoxic effects of the HEEA fraction at high concentrations. As shown in Table 2, HEH fraction contained the halogenated compound already mentioned in the previous section (malyngamide L), which was the most cytotoxic. Given the achieved results, the *A. armata* extract (HE) and HEW fraction could be safer to use in skincare products but always depending on the concentrations employed.

On the other hand, inflammation is the hallmark of the acne vulgaris cascade, and each preceding factor plays a role in triggering the inflammatory response. This includes the induction of pro-inflammatory molecules and activated pathways by *C. acnes*, hormonal imbalances, alterations in sebum composition, and cytokine secretion resulting from hyperkeratinization (reviewed in [3]). NO is a free radical that, when produced in excess, interacts with superoxide, giving rise to additional reactive nitrogen species, namely peroxynitrite [66]. It is a pro-inflammatory mediator that triggers inflammation, participating in immune responses through cytokine-activated macrophages, which release NO in elevated amounts [67]. Therefore, the potential anti-inflammatory properties of *A. armata* extracts and fractions were evaluated at lower cytotoxic concentrations by the determination of NO on macrophages. Under such conditions, a significant decrease in NO production was observed (Figure 5), indicating the efficiency of both the crude extract (HE) and the aqueous fraction (HEW) as anti-inflammatory agents. Mellouk et al. [68] found that hydroethanolic extracts from *A. taxiformis* (Rhodophyta) resulted in 21.6% NO inhibition, and Ferreira et al. [69] reported inhibition of approximately 30% with a hydroethanolic extract of *Grateloupia turuturu* (Rhodophyta), and 55% NO inhibition with a hydroethanolic extract from *Porphyra umbilicalis* (Rhodophyta). The results of these studies are consistent with the present research, although the current findings demonstrate even higher NO inhibition values, with the crude extract (HE) ranging from 57.1% to 68.7% and the HEW fraction ranging from 20.7% to 64.6%. The authors conclude that algal extracts with antioxidant compounds, such as flavonoids or other phenolic compounds, compete with oxygen to scavenge nitric oxide, thereby inhibiting nitrite production [38,70], as corroborated here for HE and HEW.

According to some studies, the fatty acid composition of *A. armata* includes saturated fatty acids (SFAs, such as palmitic and myristic acids) and mono- and poly-unsaturated fatty acids (MUFAs and PUFAs, such as palmitoleic, oleic, arachidonic, eicosapentaenoic acids, among others) [28,71]. In accordance, the organic fractions (HEH and HEEA) showed a variety of SFAs, MUFAs, and PUFAs, including palmitelaidic acid (a trans fatty acid), with SFAs being the most abundant class (Figure 6). In a study by Pinto et al. [28], palmitic acid predominated in the lipophilic extracts of *A. armata*, as occurred in the current study. Subsequent to palmitic acid, and in descending order, myristic, palmitelaidic, oleic, eicosapentaenoic, linoleic, and arachidonic acids were identified, supporting earlier research works [28,71,72,73]. As saturated and polyunsaturated fatty acids exhibit antibacterial [57,74,75,76], antioxidant [74,77], and anti-inflammatory [74,78,79] properties, they may be valuable resources in addressing the multi-factorial nature of acne vulgaris. Importantly, the quantification of esterified lipids would reveal the proportion of other fatty acids in *A. armata*, since the phospholipids identified in this study in all extracts contained very different fatty acids from those identified in the free fatty acid analysis, which may further contribute to the bioactivities.

The compounds of *A. armata* fractions were analyzed via LC-MS, since studies regarding the chemical composition of this species are limited. Several families of chemically diverse molecules were identified, including phospholipids, fatty acids, glycosylated metabolites, halogenated compounds, glycerolipids, and flavonoids. Pinto et al. [28] conducted GC-MS and UHPLC-MS profiling of *A. armata* extracts. While their work included hydroethanolic extraction (from fresh algal biomass), no halogenated compounds were detected. However, eight halogenated compounds were identified in a lipophilic extract using maceration with dichloromethane [28].

Bromine was identified as the primary component, consistent with the well-known high bromine content of the *Asparagopsis* genus, typically linked to the presence of bromoform [17]. Although chemical analyses did not detect bromoform, the elevated bromine levels in the crude extract (144 mg·L^−1^) suggested the presence of various brominated compounds not possible to identify. Halogenated compounds are associated with the seaweed’s chemical warfare and invasive success [80] and vast antimicrobial properties [81]. Element profiling revealed that *A. armata* extracts and fractions were predominantly rich in bromine (Br), chlorine (Cl), and sodium (Na), aligning with prior studies [82]. Compared with other studies, the present investigation demonstrated lower concentrations of sodium, potassium, calcium, magnesium, and phosphorus, which may arise from differences in sampling locations [83,84].

The analysis of different fractions of *A. armata* revealed variations in their biological activities concerning the tested concentrations. The crude extract (HE) exhibited high antioxidant activity (280.3 ± 7.3 µmol TE·g sample^−1^) but also demonstrated considerable cytotoxicity at higher concentrations, limiting its applicability in cosmetic products due to potential adverse effects on skin cells. Similarly, the HEH fraction showed intermediate antioxidant activity (249.5 ± 6.2 µmol TE·g sample^−1^) and significant antibacterial activity, especially against skin pathogens, as evidenced by its MIC value (5.0 × 10^−1^ mg·mL^−1^). However, it also exhibited higher cytotoxicity compared to the HEW fraction at higher concentrations. On the other hand, the HEEA fraction stood out with the highest antioxidant activity (465.2 ± 3.2 µmol TE·g sample^−1^), attributed to the presence of halogenated compounds and phospholipids, while showing lower cellular toxicity, making it promising for dermatological cosmetic applications. The HEW fraction, although demonstrating the lowest antioxidant activity (242.8 ± 3.8 µmol TE·g sample^−1^), proved to be the safest in terms of cytotoxicity, with high rates of cell viability at the tested concentrations. These observations underscore the complexity in selecting the ideal fraction for cosmetic formulations, where characteristics such as effective and safe concentrations of antioxidant, antibacterial, cytotoxic, and anti-inflammatory activities must be carefully considered.

## 4. Materials and Methods

### 4.1. Seaweed Collection and Extraction

The red macroalgae *A. armata* (gametophyte phase) were collected by scuba diving at Berlengas Natural Reserve, Portugal (41.054826, −8.656865), in July 2019. The biomass was kept in net bags and carefully transported to the laboratory within an hour, washed with fresh water, and the epibionts were removed. Then, it was dried in a ventilated incubator at 35 °C, ground to powder (0 to < 0.5 mm), and stored under vacuum in the dark at room temperature.

**Figure 7:**
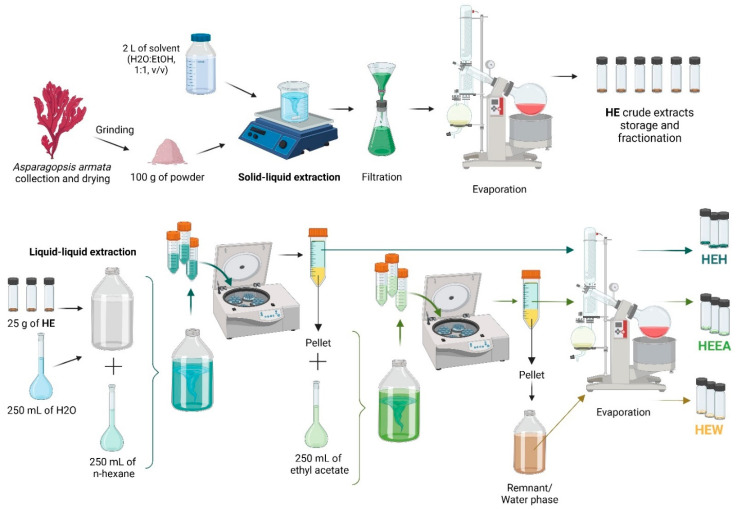
Schematics of solid–liquid and liquid–liquid extractions performed in the study and the resulting samples. Created using BioRender.

Crude solid–liquid extraction was achieved by mixing 100 g of *A. armata* powder onto 2 L of a solvent (water–ethanol 1:1, *v*/*v*) under constant magnetic stirring, protected from light, at room temperature for 2 h in triplicate (Figure 7). Extracts were filtered using filter papers (12–15 µm), evaporated under reduced pressure in a rotary evaporator at 35 °C, and desiccated at room temperature (Vacufuge^®^ plus; Eppendorf, Hamburg, Germany). Dried crude “HE” extract was weighed, and the yield was calculated (g extract·g biomass^−1^); then, a portion of each replicate was resuspended in sterile ultrapure water to achieve a stock concentration of 100 mg·mL^−1^, which was stored at −20 °C until further use in bioactivity assays. A pool of a portion from each of the three replicates of HE was prepared prior to liquid–liquid extraction (LLE). Sequential LLE was accomplished by dissolving 25 g of HE in sterile ultrapure water (100 mg·mL^−1^) (Figure 7). An equal volume of n-hexane (Carlo Erba, Cornaredo Italy) was added, mixed, separated via centrifugation (3220× *g*, 10 min, 25 °C), and the process was repeated five times. The collected volume of extraction solvent was evaporated and desiccated in the same manner as the crude extract, resulting in the n-hexane “HEH” fraction. An equal volume of ethyl acetate (VWR chemicals, Radnor, PA, USA) was added to the aqueous phase, being subjected to the same method as n-hexane, resulting in the ethyl acetate “HEEA” fraction. The remaining aqueous phase was also evaporated and desiccated, resulting in the “HEW” water fraction. Fractions were weighed and yield calculated (g fraction·g crude extract^−1^) (Figure 7). Organic fractions were resuspended in 50% (*v*/*v*) aqueous dimethyl sulfoxide (DMSO; Sigma-Aldrich, Darmstadt, Germany), and the aqueous fraction was resuspended in ultrapure sterile water only, until the same concentration was achieved. Extracts and fractions were kept frozen (−20 °C) at stock concentrations until further use.

### 4.2. Antioxidant Activity

The ORAC assay was employed to determine the extracts’ and fractions’ antioxidant activity, following a previously employed method [70]. A stock solution of Trolox (VWR, Radnor, USA) was prepared at 1 mM, and subsequent dilutions were performed, including 100, 80, 60, 40, 20, and 0 μM, intended for use as standard curves. The extracts and fractions were tested at 100 µg·mL^−1^ and diluted in PBS. A 1 mM fluorescein (Sigma-Aldrich, Darmstadt, Germany) solution was prepared, along with a 20 mM AAPH reagent (2,20-Azobis(2-methylpropionamidine) dihydrochloride (Sigma-Aldrich, Darmstadt, Germany). In a 96-well black microplate (Greiner, Kremsmünster, Austria), 120 μL of fluorescein (60 nM), 20 μL of the samples (Trolox (100 μM), extracts, fractions, or PBS), and 60 μL of AAPH pre-heated at 37 °C were added to each well, and fluorescence was read for up to 4 h with 1 min intervals at an excitation wavelength of 485 nm and an emission wavelength of 525 nm in a microplate reader (Synergy H1, Biotek, Winooski, VT, USA) at 37 °C. The results are expressed as micromoles of Trolox equivalents per gram of extract/fraction (μmol TE·g ext^−1^) and represent the mean of three independent assays with standard deviation.

### 4.3. Antimicrobial Susceptibility Testing

The *Cutibacterium acnes* (DSM-1897) was acquired from the Leibniz Institute DSMZ, and the culture conditions were followed according to the manufacturer’s instructions. Columbia Blood agar (CBA; Columbia agar base, Sigma-Aldrich, Germany; Defibrinated Sheep Blood, Frilabo, Oeiras, Portugal) was used as the primary nutritive culture medium for optimal growth in solid media, and Brain Heart Infusion broth (Sigma-Aldrich, Darmstadt, Germany) supplemented with 2% glucose (Merck, Darmstadt, Germany) was used to obtain growth in liquid media and for AST. To determine the susceptibility of *C. acnes* to *A. armata* extracts and fractions, the microdilution technique was carried out in accordance with the Clinical and Laboratory Standards Institute (CLSI, M11-A6 [85]), with some modifications, in order to find the minimum inhibitory concentration, minimum bactericidal concentration, and the percentage of bacterial growth inhibition. Three-day-old colonies of *C. acnes* cultures grown in CBA, and under anaerobiotic conditions, were dissolved in a saline solution (0.85% NaCl; Merck Millipore, Burlington, MA, USA), and the concentration of colony-forming units (CFUs) was adjusted to 2 × 10^7^ CFU·mL^−1^, so that the inoculum concentration on the microplates was 1 × 10^6^ CFU·mL^−1^. Vancomycin (final concentration in microplate wells: 4 µg·mL^−1^; Sigma-Aldrich) was used as the positive control for bacterial growth inhibition. DMSO or water were used as the vehicle control and negative control for bacterial growth inhibition. Samples were prepared from extract and fraction stock solutions (100 mg·mL^−1^) and tested at several concentrations: 1.0 × 10^−4^, 1.0 × 10^−3^, 1.0 × 10^−2^, 1.0 × 10^−1^, 2.5 × 10^−1^, 5.0 × 10^−1^, 7.5 × 10^−1^, 1, 2, 3, 4, 6, 8, and 10 mg·mL^−1^ for HE extraction in triplicate and the HEW fraction; and 1.0 × 10^−4^, 1.0 × 10^−3^, 1.0 × 10^−2^, 1.0 × 10^−1^, 2.5 × 10^−1^, 5.0 × 10^−1^, 7.5 × 10^−1^, 1, 2, 3, and 4 mg·mL^−1^ for HEH and HEEA fractions. The assay was performed using sterile round-bottom microplates (ThermoScientific, Waltham, MA, USA) for 72 h at 35 °C in an anaerobic environment provided by the Anaerocult^®^ A mini kit (Merck Millipore, Burlington, MA, USA). After the incubation period, the plates were initially examined to assess well turbidity. Wells where bacterial density was not visually observed were noted, and the lowest concentration was considered MIC. Secondly, optical density (DO) was measured at 625 nm in a microplate reader (EPOCH 2, BioTek Instruments, Winooski, VT, USA). Finally, all wells that did not exhibit apparent bacterial growth were streaked on agar plates with CBA and incubated in the same anaerobic conditions as the microplates (35 °C, 72 h). The lowest concentration where bacterial growth was not detected as a single CFU in CBA was recorded as MBC. Each condition was tested using five technical replicates and six independent assays. The results are expressed in terms of bacterial growth inhibition (percentage), and for MIC and MBC, in terms of extract or fraction mg·mL^−1^.

### 4.4. Cytotoxicity and Anti-Inflammatory Activity

#### 4.4.1. Cell Culture of HaCaT and RAW 264.7 Cell Lines

The human keratinocyte cell line HaCaT was obtained from CLS Cell Lines Service GmbH (Köln, Germany), and the mouse macrophage cell line (RAW 264.7; TIB 71) was obtained from the ATCC American Type Culture Collection (Manassas, VA, USA). Both cultures were grown and maintained in T-flasks with Dulbecco’s Modified Eagle Medium (DMEM; Sigma-Aldrich, St. Louis, MO, USA), 10% fetal bovine serum (FBS; Biowest, Nuaillé, France), Nystatin (10 U·mL^−1^; Sigma-Aldrich, St. Louis, MO, USA), Kanamycin (100 mg·L^−1^; Sigma-Aldrich, St. Louis, MO, USA), Phenol Red (Sigma-Aldrich, St. Louis, MO, USA) (only for the HaCaT culture), and they were incubated at 37 °C in CO_2_ atmosphere (5%) for up to 3 days until subculturing or plating for an assay.

To handle the HaCaT cell line, an adaptation of the CLS protocol was performed. Subculturing was achieved by removing the medium from 25 cm^2^ flasks containing a monolayer of adherent cells and washing with 3 mL of PBS. Then, 1 mL of EDTA (0.05% in PBS) was added in order to cover the cell sheet and incubated for 10 min at 37 °C. Afterward, 1 mL of trypsin from porcine pancreas (4×; Sigma-Aldrich, Germany) was added and incubated for 1 min, and cells were detached. A volume of 4 mL of supplemented DMEM was added to inactivate the enzyme, and centrifugation was performed (129× *g*, 5 min). The harvested cells were divided into three new T-flasks with fresh DMEM for subculturing or counted and seeded in microplates for further assays.

#### 4.4.2. Cytotoxicity Evaluation

Cell proliferation was assessed by employing different concentrations of *A. armata* extracts and fractions using the 3-(4,5-dimethylthiazol-2-yl)-2,5-diphenyl tetrazolium bromide (MTT) assay, according to a previous study [70]. Briefly, HaCaT and RAW 264.7 cells were seeded at 5 × 10^3^ cells· well^−1^ in a 96-well flat-bottom sterile microplate (VWR International) and incubated for 24 h (37 °C in 5% CO_2_ atmosphere). After that period, cells were treated with several concentrations of *A. armata* extracts and fractions in phosphate-buffered saline (PBS; Lonza, Switzerland): HE, HEH, HEEA, HEW at 1 × 10^−4^, 1 × 10^−3^, 1 × 10^−2^, 1 × 10^−1^, 2.5 × 10^−1^, 5.0 × 10^−1^, 7.5 × 10^−1^, 1, and 2 mg·mL^−1^ for HaCaT cells; HE and HEW at 1 × 10^−6^, 1 × 10^−5^, 1 × 10^−4^, 1 × 10^−3^, 1 × 10^−2^, 1 × 10^−1^, 2.5 × 10^−1^, 5.0 × 10^−1^, 7.5 × 10^−1^, 1, and 2 mg·mL^−1^, HEH and HEEA at 1 × 10^−4^, 1 × 10^−3^, 1 × 10^−2^, 1 × 10^−1^, 2.5 × 10^−1^, 5.0 × 10^−1^, and 7.5 × 10^−1^ mg·mL^−1^ for RAW 264.7 cells, for 24 h. Wells where cells were grown in a culture medium were used as the growth control. DMSO or water were used as the vehicle control and negative control for cell growth inhibition. DMSO was also used for cell death control. After incubation, the medium was removed, and 100 µL of the MTT solution (0.5 mg·mL^−1^ in 5% FBS DMEM without phenol red) was added to all wells in the microplate and incubated for 4 h in the dark. The MTT solution was then removed, and 100 µL of DMSO was incubated for 15 min in the dark until formazan was completely solubilized. Absorbance was measured at 570 nm using a microplate spectrophotometer (Epoch2, BioTek, Vermont, EUA). Each condition underwent testing with eight technical replicates and at least four independent assays. The results are expressed in terms of viability (percentage) by comparing the samples with both viability and death controls.

#### 4.4.3. Measurement of Nitric Oxide (NO) Production

The production of NO in RAW 264.7 cells was measured using a Griess diazotization reaction, following the method described in [70]. Microplates were seeded with 1 × 10^5^ cells·well^−1^ and incubated at 37 °C in 5% CO_2_ for 24 h. Following this, cells were exposed to all concentrations of extracts with cell viability above 75% for 6 h; afterward, they were treated with a lipopolysaccharide (LPS) solution at a final concentration of 1.5 μg·mL^−1^ for 22 h. Subsequently, 150 μL of the cell culture supernatants was mixed with 50 μL of the Griess reagent (Sigma-Aldrich, Darmstadt, Germany) and incubated for 15 min in the dark at room temperature. Absorbance was then recorded at 540 nm using a microplate spectrophotometer (Epoch2, BioTek, Vermont, EUA). Each condition was tested using six technical replicates and five independent assays. The results are expressed in NO production (%) compared with samples with LPS control (cells treated with PBS for 6 h instead of the extract solution and then stimulated with LPS).

### 4.5. Chemical Characterization of Asparagopsis armata Extracts and Fractions

#### 4.5.1. Free Fatty Acids

The identification of free fatty acids was conducted via liquid chromatography–mass spectrometry (LC-MS), using a method adapted from Saha et al. [86]: 1 mg of *A. armata* organic fractions (HEH and HEEA) was dissolved in 1 mL of dichloromethane:methanol (1:2) and filtered through a 0.22 µm size pore membrane into an amber glass vial in triplicate. The analysis of free fatty acid profiles was performed using an HPLC system (Agilent 1260 series) coupled with a Q-TOF mass spectrometer (Agilent 6520) operating in the electrospray ionization (ESI) source mode. A Poroshell 120 C18 column (2.7 µm, 3.0 × 150 mm) from Agilent was used for chromatographic separation. Mobile phase (A) consisted of 2 mM ammonium acetate in water, and mobile phase (B) contained 2 mM ammonium acetate in 95% acetonitrile. Separation was achieved through gradient elution, starting with 60% B for 1 min, followed by an increase to 90% B over 2.5 min. Subsequently, 90% B was held for 1.5 min and further increased to 100% B over 5 min. Afterward, 100% B was maintained for 4 min, then reduced to 60% B over 0.5 min, and held for 1 min before the next run. The mobile phase flow rate was 0.3 mL·min^−1^ for the first 5 min and increased to 0.6 mL·min^−1^ after 10 min, remaining constant until the end of the run. The volume of the injection was 10 µL. The mass spectrometer was operated in the negative ionization mode, searching for lipids in the *m*/*z* range of 50–1100. The parameters, including the drying gas flow rate, temperature, and nebulizer pressure, were set to 5 L·min^−1^, 325 °C, and 30 psi, respectively. The voltage of the fragmentor was maintained at 175 V, the skimmer was set to 65 V, and the capillary operated at 3500 V. The reference masses used for monitoring in the negative ion mode were 1033.988 and 112.9855.

#### 4.5.2. Compound Screening

For the untargeted identification of compounds, a procedure employing LC-MS was conducted and adapted [86]. Due to the presumably high content of salts and polymers, the aqueous fraction of HE (HEW) was first dissolved in methanol (500 mg in 1 L) for 1 h under constant magnetic stirring, followed by centrifugation (3220× *g*, 10 min, 25 °C), and the insoluble solids were discarded. The solubilized content was recovered through solvent evaporation under reduced pressure in a rotary evaporator at 35 °C and desiccated at room temperature (Vacufuge^®^ plus; Eppendorf, Hamburg, Germany), resulting in HEWm. The fractions of *A. armata* (HEH, HEEA, HEWm) crude extract (HE), which did not undergo purification, were dissolved in acetonitrile:methanol (ACN:MeOH; 60:40) at a concentration of 5 mg·mL^−1^ and filtered through a 0.22 µm size pore membrane into an amber glass vial in triplicate. LC-MS analysis was carried out using an Agilent 1260 LC system equipped with a Poroshell 120 ec-C18 column (4.6 × 150, 2.7 micron) coupled with a mass spectrometer Agilent 6520 QTOF and a diode array detector (DAD). The elution was performed at a flow rate of 0.6 mL·min^−1^ using a mobile phase consisting of 95% water +0.1% formic acid (A) and 95% acetonitrile + 0.1% formic acid (B). Gradient elution was applied as follows: 4% of phase B was held constant from 0 to 3 min, then increased to 20% from 3 to 8 min, followed by an increase to 70% of phase B from 8 to 15 min, 90% of phase B from 15 to 25 min, and finally, 100% of phase B from 25 to 40 min. The mobile phase was held constant at 100% B from 40 to 50 min. An equilibration time of 5 min was assigned to ensure the initial conditions were equilibrated before proceeding with the next analysis. Detection was carried out using DAD at wavelengths of 210, 267, 280, and 293 nm and via MS scanning in the *m*/*z* range of 50–1700. Samples were injected using 5 µL of the volume, with electrospray ionization (ESI) as the ionization type, and analyzed in both positive and negative ionization modes. The LC-MS data, including UV chromatograms, DAD spectra, and mass fragmentation patterns obtained from the QTOF, were analyzed for the detection of bioactive compounds using Metlin library search after chromatogram deconvolution. The hits list was filtered based on specific criteria (number of hits ≤ 3, score ≥ 85, absolute mass difference ≤ 5 ppm, and number of ions ≥ 5) and then carefully examined one by one. The final identification was made considering the quality of the MS-based identification proposed, the visual similarity between the hits’ mass spectra and the library, chromatographic peak definition, and evaluation of the biological and chemical relevance of the identified compounds in the context of the studied algal biomass.

#### 4.5.3. Element Profile

Element fingerprinting was performed based on a previous work, with slight modifications [87]. All labware for elemental analysis underwent decontamination in acid baths for 48 h. *A. armata* extracts and fractions were completely mineralized using 3.2 mL of 70% *v*/*v* HNO_3_ in Teflon vessels, following the EPA 3052 method (US Environmental Protection Agency (USEPA), 1995). This mineralization process was carried out through microwave-assisted extraction using the Multiwave GO system (Anton Paar GmbH, Graz, Austria). After cooling down, the acid extracts were mixed with an internal standard (Gallium) at a final concentration of 1 mg·L^−1^. For further analysis, 5 µL of each sample digestion product was applied to a silicone-coated quartz disk (Bruker Nano, Berlin, Germany) and evaporated at 60 °C on a hot plate until complete dryness was reached. Elemental concentrations were then determined using total reflection X-ray fluorescence spectroscopy (TXRF S2 PICOFOX, Bruker, Berlin, Germany). To ensure the accuracy of the results, quality control measures were adopted, including instrumental recalibration (gain correction, sensitivity analysis, and multi-elemental standards) and analytical blanks. The internal standard (Gallium) was utilized to determine individual elemental concentrations, and the experiment was carried out in triplicate.

### 4.6. Statistical Analysis

Graphical representations, descriptive statistics, one-way analysis of variance (ANOVA), and multiple comparisons tests (Dunnett’s or Tukey’s, depending on the case) were conducted in GraphPad Prism v8.4.3 (GraphPad Software, La Jolla, CA, USA), and significant differences were represented by * *p* < 0.05, ** *p* < 0.01, *** *p* < 0.001, **** *p* < 0.0001, with a consistent level of significance set at 0.05 for all statistical tests. Residual plots were employed to assess model assumptions, including normality and homoscedasticity.

## 5. Conclusions

The exploration of *Asparagopsis armata* for skincare applications is an important contribution to sustainability in the cosmetic industry. As the demand for eco-friendly cosmetics rises, leveraging natural resources like *A. armata* addresses consumer preferences and environmental concerns. This study employed industry-friendly biomass extraction methods that maximize yield and compound recovery, aligning with biorefinery and circular economy principles.

The results demonstrate the bioactive potential of *A. armata* extracts, particularly in combating acne vulgaris, showcasing antioxidant, antibacterial, and anti-inflammatory properties. Safety analyses on human keratinocytes and murine macrophages indicate the dose-specific safety of the extracts, allowing for future performance comparisons.

While the HE extract and its fractions exhibit notable bioactivity, their effective and safe concentrations do not always coincide, limiting their direct use in functional cosmetics. In contrast, the HEW fraction shows a favorable balance of biological activities with low cytotoxicity, inhibiting *Cutibacterium acnes* growth at 1.0 × 10^−4^ mg·mL^−1^ while maintaining non-cytotoxicity and potential anti-inflammatory effects. This addresses the challenge of inhibiting *C. acnes* growth without compromising human cell viability.

The chemical analysis reveals a diverse array of compounds present in *A. armata* fractions, highlighting its chemical complexity and potential for further exploration, comprising various compounds, such as phospholipids, glycosylated metabolites, flavonoids, and fatty acids, alongside high bromine, sodium, and chlorine content.

Overall, *A. armata* extracts hold promise as natural anti-acne ingredients in topical formulations. While this study enhances our understanding of *A. armata*’s bioactive profile, further research is needed to elucidate its therapeutic potential, identify active compounds and their mechanisms, and explore economically feasible utilization strategies for wild-caught invasive biomass, including seasonal and geographical variability and sustainable harvesting practices.

## Figures and Tables

**Table 1:** Extractions performed on *Asparagopsis armata* biomass and associated yields expressed in dry weight percentage.

Water/Ethanol (50:50 *v*/*v*)	Yield % (m/m) ^1^	Water/Solvent (50:50 *v*/*v*)	Yield % (m/m) ^2^
HE R1	15.01%	HEH	2.97%
HE R2	13.15%	HEEA	4.09%
HE R3	13.92%	HEW	92.94%

HE—Hydroethanolic solid–liquid crude extraction; HEH—Liquid–liquid sequential extraction from HE with n-hexane; HEEA—Liquid–liquid sequential extraction from HE with ethyl acetate; HEW—Liquid–liquid sequential extraction from HE of the aqueous remnant; ^1^ Yield calculated from the ratio grams of extract obtained from grams of powdered *A. armata*; ^2^ Yield calculated from the ratio grams of fraction obtained from grams of *A. armata* hydroethanolic crude extracts.

**Table 2:** Compounds tentatively identified in the n-hexane fraction (HEH) of *Asparagopsis armata* crude extract (HE), as determined by LC-MS. RT—Retention time (in minutes); *m*/*z*—Mass-to-charge ratio; PI—Phosphatidylinositol.

Compound	RT	*m*/*z*	Calc. *m*/*z*	|Diff| (ppm)	Mass	Formula	Mode
2-Hydroxyethanesulfonate	2.437	124.9913	[M − H]^−^ 124.9914	0.80	125.9986	C2 H6 O4 S	-
Malyngamide L	9.944	485.3123	[M + NH_4_]^+^ 485.3152	5.89	467.2782	C26 H42 Cl N O4	+
PI (13:0/12:0)	10.192	713.4203	[M − H]^−^ 713.4247	6.17	712.4131	C34 H65 O13 P	+
3,11-dihydroxymyristic acid	14.924	259.1897	[M − H]^−^ 259.1915	6.94	260.1969	C14 H28 O4	-
Prostaglandin F2α	29.667	473.2763	[M + HCOO]^−^ 473.2745	3.80	428.2789	C23 H40 O7	-
(3xi,6E)-1,7-Diphenyl-6-hepten-3-ol	31.458	266.1657	[M●]^−^ 265.1598	7.14	266.1681	C19 H22 O	-

**Table 3:** Compounds tentatively identified in the ethyl acetate fraction (HEEA) of *Asparagopsis armata* crude extract (HE), as determined by LC-MS. RT—Retention time (in minutes); *m*/*z*—Mass-to-charge ratio; PG—Phosphatidylglycerol; PA—Phosphatidic acid; MGDG—Monogalactosyldiacylglycerol.

Compound	RT	*m*/*z*	Calc. *m*/*z*	|Diff| (ppm)	Mass	Formula	Mode
Galactosylglycerol	2.469	277.0907	[M + Na]^+^ 277.0894	4.69	254.1012	C9 H18 O8	+
O-Succinyl-L-homoserine	6.797	237.1072	[M + NH_4_]^+^ 237.1092	8.44	219.0734	C8 H13 N O6	+
PA (22:1(11Z)/0:0))	21.487	491.3140	[M − H]^−^ 491.3132	1.63	492.3212	C25 H49 O7 P	-
1,2-di-(9Z,12Z,15Z-octadecatrienoyl)-3-(8-(2E,4Z-decadienoyloxy)-5,6-octadienoyl)-sn-glycerol	25.19	918.6828	[M + NH_4_]^+^ 918.6817	1.20	900.6489	C57 H88 O8	+
PA (20:5(5Z,8Z,11Z,14Z,17Z)/22:6(4Z,7Z,10Z,13Z,16Z,19Z))	27.129	767.4639	[M + H]^+^ 767.4646	0.91	766.4578	C45 H67 O8 P	+
Prostaglandin F2α	29.812	473.2754	[M + HCOO]^−^ 473.2745	1.90	428.2776	C23 H40 O7	-
PG (P-20:0/22:2(13Z,16Z))	34.244	860.6757	[M + NH_4_]^+^ 860.6734	2.67	842.6406	C48 H91 O9 P	+
PA (21:0/22:2(13Z,16Z))	34.809	816.6504	[M + NH_4_]^+^ 816.6477	3.31	798.6156	C46 H87 O8 P	+
MGDG (16:0/18:2(9Z,12Z))	35.455	772.5951	[M + NH_4_]^+^ 772.5933	2.33	754.5601	C43 H78 O10	+

**Table 4:** Compounds tentatively identified in the methanol fraction of remnant aqueous fraction (HEWm) of *Asparagopsis armata* crude extract (HE), as determined by LC-MS. RT—Retention time (in minutes); *m*/*z*—Mass-to-charge ratio; PI—Phosphatidylinositol; DG—Diacylglycerol.

Compound	RT	*m*/*z*	Calc. *m*/*z*	|Diff| (ppm)	Mass	Formula	Mode
PI (O-16:0/16:0)	19.033	814.5822	[M + NH_4_]^+^ 814.5804	2.21	796.5479	C41 H81 O12 P	+
DG (24:1(15Z)/22:6(4Z,7Z,10Z,13Z,16Z,19Z)/0:0)	27.737	751.6254	[M + H]^+^ 751.6235	2.53	750.6177	C49 H82 O5	+
Gingerglycolipid A	29.63	677.3760	[M + H]^+^ 677.3743	2.51	676.3689	C33 H56 O14	+

**Table 5:** Element profile of the hydroethanolic extract (HE), n-hexane fraction (HEH), ethyl acetate fraction (HEEA), and remnant aqueous fraction (HEW) of *Asparagopsis armata* as mass percentage of the dry extract.

Element	HE (MPDE)	HEH (MPDE)	HEEA (MPDE)	HEW (MPDE)
Br	4.01 ± 0.30	0.45 ± 0.05	1.47 ± 0.08	4.35 ± 0.31
Cl	2.25 ± 0.56	0.045 ± 0.02	0.11 ± 0.01	1.98 ± 0.15
Na	1.56 ± 0.29	1.80 ± 0.17	0.82 ± 0.14	1.72 ± 0.39
K	0.52 ± 0.05	0.034 ± 0.01	0.06 ± 0.01	0.56 ± 0.05
Ca	0.32 ± 0.03	0.01 ± 0.01	0.04 ± 0.01	0.33 ± 0.02
S	0.28 ± 0.06	0.17 ± 0.02	0.07 ± 0.01	0.24 ± 0.01
I	0.22 ± 0.06	0.27 ± 0.03	0.95 ± 0.04	0.20 ± 0.03
Mg	0.08 ± 0.03	0.05 ± 0.05	0.01 ± 0.00	0.11 ± 0.02
P	0.03 ± 0.04	0.01 ± 0.01	0.02 ± 0.00	0.01 ± 0.02

The values represent the mean ± standard deviation. Mass percentage of the dry extract ((mg element·mg extract^−1^) × 100); Br—Bromine; Cl—Chlorine; Na—Sodium; K—Potassium; Ca—Calcium; S—Sulfur; I—Iodine; Mg—Magnesium; P—Phosphorus.

## Data Availability

The original contributions presented in this study are included in the article. Further inquiries can be directed to the corresponding author(s).
